# Comment on Naef, R.; Acree, W.E., Jr. Revision and Extension of a Generally Applicable Group-Additivity Method for the Calculation of the Standard Heat of Combustion and Formation of Organic Molecules. *Molecules* 2021, *26*, 6101

**DOI:** 10.3390/molecules28176215

**Published:** 2023-08-24

**Authors:** Robert J. Meier

**Affiliations:** Independent Researcher, 52525 Heinsberg, Germany; r.meier@planet.nl

Next to the paper referred to in the title [1], Naef and Acree have published a series of papers involving the group contribution (GC) method applied to a larger variety of physico-chemical properties [2,3,4,5,6,7]. The GC method is based on the assumption that some aspects of chemical groups are the same in many different molecules. The GC method is a so-called data-driven model with experimental data being used to parametrize the model. When a molecular property of interest is the sum (additive) of the individual properties of the molecular fragments j, for the heat of formation ΔH_f_, the equation reads:

(1) $$\Delta H_{f}=\underset{j=1,N}{\sum }\mathrm{Nj}\cdot \Delta H_{f}\left(j\right)$$

We talk more specifically about a group additivity method, as in the title of the paper by Naef and Acree.

What is greatly surprising is that Neaf and Acree have not referred to very many relevant publications over the last 25 years and suggest that they report new results since Benson and Cohen [8,9], who reported their approach in the years 1993 and 1996; they are the only references to the GC approach they have referred to. Moreover, Naef and Acree wrote [2] ‘Most of these various approaches have been optimized for a certain class of compounds and are therefore not generally applicable. In contrast, the present calculation method is easily extendable and in principle enables the calculation of the heat of combustion and formation of literally any organic molecule under the sun’. The suggestion that the paper by Naef and Acree is the first one with the capability to treat ‘any molecule under the sun’ is simply totally incorrect. There have been numerous papers by several excellent groups that have reported significant progress on this topic over the last 25 years, viz. [10,11,12,13,14,15,16,17,18,19,20,21,22,23,24,25,26,27,28,29,30], and this list is definitely not exhaustive; moreover, the methods reported in this paper can be applied to an extremely large number of molecules (no method is applicable to all molecules under the sun as this would require GC parameters for all molecules under the sun). In Ref. [7] (Neaf and Acree), reference to GC methods are only the previous publications by Naef and Acree. Many of the properties mentioned in their papers have been treated using GC methods in the meantime, see more specifically the thesis by Amol Shivajirao Hukkerikar (2013), which is freely available [31], and Hukkerikar’s results have also been published in scientific papers, including references [19,20,24]. From the abstract of Hukkerikar’s thesis, we quote ‘In total, 21 thermo-physical properties and 22 environmental-related properties of pure components which include normal boiling point, critical constants, standard enthalpy of formation, liquid viscosity, fathead minnow 96 h LC50, oral rat LD50, global warming potential, emission to urban air (carcinogenic and noncarcinogenic) among others are modeled and analyzed. For all the estimated pure component properties, the corresponding 95% confidence intervals are also reported thereby providing information on the degree of accuracy of the property estimates’. Furthermore ‘is illustrated by considering performance improvement of models for enthalpy of formation, enthalpy of fusion, and critical temperature. For all properties listed above, it has been possible to achieve significant improvements in the performance of their models’. Moreover, returning to the statement by Naef and Acree that up till now methods have not allowed for the evaluation of properties for any molecule under the sun, a comprehensive software suite exists and has been actively maintained (DTU, Danish Technical University) for very many years now involving a molecule drawing tool and the subsequent instantaneous generation of a larger variety of physico-chemical properties [32]. There are many more programs (which have also been available for many years now) which predict various specific properties, e.g., log Kow, toxicity, etc., with very many of these being freely available on the Internet (we will not reference any of these here, but they can be easily found for a specific property).

When talking about the heat of formation, Naef and Acree reported that ‘the standard error is still 18.14 kJ/mol’, and that there is species for which the difference between the experiment and the model is as high as 55 kJ/mol, which is very much beyond the ‘chemical accuracy’ (historically defined as 1 kcal/mol, and thus 4 kJ/mol, for the difference between the model and experimental value) needed for proper process design [24,26]. Such large deviations describe chemical equilibira extremely poorly. This is truly ill performance compared to various studies reported before, e.g., the work reported in Ref. [24], which quoted ‘an average absolute error of 1.75 kJ/mol and standard deviation of 2.61 kJ/mol’.

A definite further issue regarding the quality of the analysis and model by Naef and Acree is that they have simply taken the experimental heat of formation data from a very large number of different papers, and their papers contain a corresponding very large number of references. To obtain accurate and reliable predictions by a data-driven model, one requires accurate and consistent experimental data. This is definitely not the case when data are collected from a large number of references without any discussion of the quality, where, with quality, we refer to the accuracy and reliability of the numerical values. For many compounds, one can find different values in the literature, with differences going beyond chemical accuracy (4 kJ/mol), and therefore the selection of sufficiently reliable data is of crucial importance [26,27,28,29] to obtain a model with good and reliable predictability. In this context, Naef and Acree wrote ‘However, contrary to the approach in the earlier paper, a further restriction was introduced in that only those compounds were allowed to remain in the consecutive calculations, the experimental heat of formation values of which did not deviate by more than three times the cross-validated standard error from the cross-validated calculated value. Accordingly, the final group contributions rested on 5030 compounds’, which clearly suggests that compounds that were too far away were simply omitted following a fully automated procedure. This, however, (i) does not guarantee that all the other experimental data are correct and that the predictions are therefore correct and should be retained, and (ii) it does not exclude that the model is simply not correct and that the compounds were removed as a result of that. Experience has demonstrated that only by adopting truly reliable data can one find high-quality models, see, e.g., Refs. [14,15,16,22,26,27,28,29,30].

So, it seems justified to state that Naef and Acree claim new and better results compared to what they referred to as the existing literature, whereas they have simply ignored a larger number of excellent scientific publications in the period after 1996, which seems astonishing as this concerns a quarter of a century. Earlier works, i.e., before Naef and Acree published their paper, have shown that approaches other than purely atom-based groups are more successful for accounting for the heat of formation with a good accuracy [10,11,24], and, more recently, even up to chemical accuracy (1 kcal/mol), with none or exceptionally few outliers [26,27,28,29].
